# Molecular adsorbent recirculating system and single-pass albumin dialysis in liver failure – a prospective, randomised crossover study

**DOI:** 10.1186/s13054-015-1159-3

**Published:** 2016-01-04

**Authors:** Christoph Sponholz, Katja Matthes, Dina Rupp, Wolf Backaus, Sebastian Klammt, Diana Karailieva, Astrid Bauschke, Utz Settmacher, Matthias Kohl, Mark G. Clemens, Steffen Mitzner, Michael Bauer, Andreas Kortgen

**Affiliations:** 1 Department of Anaesthesiology and Critical Care Medicine, Jena University Hospital, Erlanger Allee 101, 07747 Jena, Germany; 2 Charité Research Organisation, Berlin, Germany; 3 Institute of Clinical Chemistry and Laboratory Diagnostics, Jena University Hospital, Jena, Germany; 4 Center for Sepsis Control and Care, Integrated Treatment and Research Center, Jena University Hospital, Jena, Germany; 5 Division of General, Visceral and Vascular Surgery, Jena University Hospital, Jena, Germany; 6 Department of Medical and Life Sciences, Furtwangen University, Villingen-Schwenningen, Germany; 7 The Liver-Biliary-Pancreatic Center, Carolinas Medical Center, Charlotte, NC USA; 8 Department of Biology, University of North Carolina at Charlotte, Charlotte, NC USA; 9 Division of Nephrology, Department of Medicine, Rostock University Medical Centre, Rostock, Germany; 10 Fraunhofer Institute for Cell Therapy and Immunology, Extracorporeal Immunomodulation Project Group, Rostock, Germany

**Keywords:** Extracorporeal liver support, Liver failure, Albumin dialysis, Bile acid, Albumin-binding capacity

## Abstract

**Background:**

The aim of extracorporeal albumin dialysis (ECAD) is to reduce endogenous toxins accumulating in liver failure. To date, ECAD is conducted mainly with the Molecular Adsorbents Recirculating System (MARS). However, single-pass albumin dialysis (SPAD) has been proposed as an alternative. The aim of this study was to compare the two devices with a prospective, single-centre, non-inferiority crossover study design with particular focus on reduction of bilirubin levels (primary endpoint) and influence on paraclinical and clinical parameters (secondary endpoints) associated with liver failure.

**Methods:**

Patients presenting with liver failure were screened for eligibility and after inclusion were randomly assigned to be started on either conventional MARS or SPAD (with 4 % albumin and a dialysis flow rate of 700 ml/h). Statistical analyses were based on a linear mixed-effects model.

**Results:**

Sixty-nine crossover cycles of ECAD in 32 patients were completed. Both systems significantly reduced plasma bilirubin levels to a similar extent (MARS: median −68 μmol/L, interquartile range [IQR] −107.5 to −33.5, *p* = 0.001; SPAD: −59 μmol/L, −84.5 to +36.5, *p* = 0.001). However, bile acids (MARS: −39 μmol/L, −105.6 to −8.3, *p* < 0.001; SPAD: −9 μmol/L, −36.9 to +11.4, *p* = 0.131), creatinine (MARS: −24 μmol/L, −46.5 to −8.0, *p* < 0.001; SPAD: −2 μmol/L, −9.0 to +7.0/L, *p* = 0.314) and urea (MARS: −0.9 mmol/L, −1.93 to −0.10, *p* = 0.024; SPAD: −0.1 mmol/L, −1.0 to +0.68, *p* = 0.523) were reduced and albumin-binding capacity was increased (MARS: +10 %, −0.8 to +20.9 %, *p* < 0.001; SPAD: +7 %, −7.5 to +15.5 %, *p* = 0.137) only by MARS. Cytokine levels of interleukin (IL)-6 and IL-8 and hepatic encephalopathy were altered by neither MARS nor SPAD.

**Conclusions:**

Both procedures were safe for temporary extracorporeal liver support. While in clinical practice routinely assessed plasma bilirubin levels were reduced by both systems, only MARS affected other paraclinical parameters (i.e., serum bile acids, albumin-binding capacity, and creatinine and urea levels). Caution should be taken with regard to metabolic derangements and electrolyte disturbances, particularly in SPAD using regional citrate anti-coagulation.

**Trial registration:**

German Clinical Trials Register (www.drks.de) DRKS00000371. Registered 8 April 2010.

**Electronic supplementary material:**

The online version of this article (doi:10.1186/s13054-015-1159-3) contains supplementary material, which is available to authorized users.

## Background

Liver failure—acute or acute-on-chronic—is associated with high mortality. According to the toxin hypothesis of liver failure, extracorporeal albumin dialysis (ECAD) may provide a therapeutic option during critical care by reducing endogenous albumin-bound toxic agents such as bile acids [1]. Currently, ECAD is mainly conducted using the Molecular Adsorbents Recirculating System (MARS). Several clinical trials have certified MARS as a feasible tool in reducing patients’ bilirubin levels, improving haemodynamic status and hepatic encephalopathy (HE) [2–4]. However, large clinical trials have failed to demonstrate a survival benefit for patients treated with MARS [2, 4]. Another ECAD technique, single-pass albumin dialysis (SPAD), has been proposed. This technique enables easy access to extracorporeal liver support by using standard dialysis devices [5]. In case reports and small clinical studies, researchers have reported the feasibility of SPAD in reducing patients’ bilirubin levels and improving their clinical condition (i.e., HE) [6]. However, while MARS is routinely prepared with 600 ml of a 20 % albumin solution according to manufacturers’ instructions, SPAD applications were reported to run on different albumin concentrations and dialysate flow rates [7, 8]. Thus, comparison of both techniques requires clear definitions of SPAD construction. Recently, our study group retrospectively compared the effects of MARS and 4 % SPAD with a dialysis flow rate of 700 ml/h on clinical and laboratory parameters in critically ill patients [9]. Similar settings have already been used in an in vitro comparison of MARS and SPAD that resulted in equivalent detoxification [8]. Among others, in our retrospective study, both devices were comparable in reducing bilirubin levels, but MARS treatment resulted in higher renal dialysis capacity, reflected by lower creatinine and urea levels after treatment [9]. Moreover, the equivalence of both devices in clinical routine was questioned, as chemical stabilizers added to albumin solutions may reduce performance, especially during SPAD, while they are partially removed by recirculation and purification in the MARS device [10, 11].

Thus, the aim of this study was to prospectively compare the performance of MARS and 4 % SPAD treatment in patients with severe liver failure using a single-centre crossover design, with particular focus on clinical and laboratory parameters. As a primary endpoint, we hypothesised non-inferiority in bilirubin reduction of SPAD in comparison to MARS. Changes in laboratory and clinical liver-specific parameters in both devices constituted secondary study endpoints.

## Methods

### Study design

The study was designed as a single-centre, randomised, controlled, replicated crossover study. On the basis of our retrospective study comparing the efficacy of MARS and SPAD treatment [9], power analysis revealed 64 extracorporeal liver support cycles to assess non-inferiority of SPAD in reducing bilirubin levels in comparison to MARS treatment. This parameter served as the primary endpoint in the present analysis. Calculated with a 10 % dropout rate, 70 liver support crossover cycles were planned for patient recruitment. Thus, patients were randomised to start on either MARS followed by SPAD treatment the following day or vice versa. If extracorporeal liver support was deemed necessary in a patient after the first crossover cycle, the patient could be randomised again for up to four crossover cycles. The study was approved by the ethics committee of Friedrich-Schiller University Hospital, Jena, Germany (ID 2435-12/08) and registered at German Clinical Trials Register (www.germanctr.de) (DRKS00000371), where the study protocol can be found.

### Patient recruitment

All patients in the surgical intensive care unit of Jena University Hospital with diagnoses of acute liver failure, acute-on-chronic liver failure or liver graft failure were screened for study inclusion if ECAD was indicated by the intensivist in charge. According to the department’s standard operating procedure (SOP), ECAD was considered when patients with a pertinent diagnosis presented the following signs and symptoms:

1. Plasma disappearance rate of indocyanine green (PDR_ICG_) <8–10 %/minute, and
2. Plasma bilirubin level >170 μmol/L, and
3. International normalised ratio (INR) >1.5 and/or
4. Symptoms of HE grade II or higher

Patients younger than 18 years of age were not eligible for study inclusion. After written informed consent was obtained from the patients or their legal representatives, patients were randomised for ECAD via sealed opaque envelopes to one of the study arms. Block randomisation was performed with blocks of 4, 6 or 8.

### ECAD procedure and blood sampling

Vascular access was obtained via a triple-lumen haemodialysis catheter (Trilyse Expert; Vygon, Aachen, Germany) introduced into the femoral, jugular or subclavian vein. MARS and SPAD devices were built up as described previously [9]. Depending on patients’ haemodynamics, blood flow rates were set at between 100 and 150 ml/minute in both systems, with equal flow rates in the corresponding crossover treatments. The MARS monitor (Gambro, Lund, Sweden) was attached to a standard haemodialysis machine (BM25; Edwards Lifesciences, Unterschleissheim, Germany) and planned to run for a duration of 8 h according to the manufacturer’s instructions. Albumin flow rates were equalized to blood flow rates, and dialysis flow rates were adjusted to 2000 ml/h. For SPAD (multiFiltrate; Fresenius Medical Care, Bad Homburg, Germany), dialysis flow rates were set to 700 ml/h. One thousand millilitres of fluid was removed from a 5000-ml dialysis solution bag (Ci-Ca Dialysate K2 *P*lus in case of regional citrate anti-coagulation or multiBic dialysate for heparin anti-coagulation; Fresenius Medical Care) and replaced with 1000 ml of 20 % albumin solution (CSL Behring, Marburg, Germany) containing 19.2 g of human albumin; 125 mmol/L Na^+^; maximum 100 mmol/L Cl^−^, HCl or NaOH for pH adjustment; 16 mmol/L caprylate; and 16 mmol/L *N*-acetyl-d,l-tryptophan. This resulted in a final human albumin concentration of 4 %. Using a 5000-ml dialysis solution bag, the flow rate of 700 ml/h resulted in a treatment cycle of about 7 h. Blood anti-coagulation was maintained either using regional citrate application or by systemic infusion of unfractionated heparin. In case of regional citrate anti-coagulation citrate (4 % sodium citrate; Fresenius Kabi, Bad Homburg, Germany) was applied before the haemofilter with the aim of achieving a final ionised postfilter calcium level of 0.25–0.35 mmol/L, followed by calcium reversal (1 N calcium chloride solution; Serumwerk Bernburg AG, Bernburg, Germany). In the absence of bleeding tendency and/or ongoing heparin anti-coagulation, infusion rates of unfractionated heparin were adjusted to a final activated clotting time of 140–200 seconds. Blood sampling and measurement of clinical or paraclinical parameters were carried out no more than 30 minutes before or after ECAD, respectively. Blood samples for routine laboratory parameters were immediately sent to the clinical laboratory for measurement. Plasma samples for non-routine measurements were immediately centrifuged (4700 U/minute, 4 °C) for 10 minutes. Serum samples were centrifuged (4700 U/minute, 4 °C) for 10 minutes after 30 minutes of resting. All samples were kept frozen at −80 °C until evaluation.

### Clinical assessment

Haemodynamic parameters (i.e., arterial pressure, heart rate and central venous pressure [CVP]), were recorded before and after each ECAD cycle. Cardiac output, intrathoracic blood volume index (ITBI), extravascular lung water index (ELWI) and systemic vascular resistance index were recorded if extended haemodynamic monitoring was implemented, using transpulmonary thermodilution (PiCCO; PULSION Medical Systems, Feldkirchen, Germany) [12]. PDR_ICG_ was assessed after intravenous injection of 0.25–0.5 mg/kg body weight of indocyanine green using pulse dye densitometry (LiMON; PULSION Medical Systems) [13]. HE grade using the Hepatic Encephalopathy Scoring Algorithm (HESA) [14] and the Glasgow Coma Scale score [15] and Ramsay score [16] were evaluated to detect neurological alterations. Acute Physiology and Chronic Health Evaluation II, Simplified Acute Physiology Score II and Sequential Organ Failure Assessment scores were assessed daily to evaluate illness severity and organ dysfunction [17].

### Cytokine measurement

Bio-Plex Pro Human Cytokine 8-plex Assay (Bio-Rad Laboratories, Hercules, CA, USA) was used to detect human cytokines interleukin (IL)-2, IL-4, IL-6, IL-8, IL-10, granulocyte-macrophage colony-stimulating factor, interferon-γ and tumour necrosis factor-α according to the manufacturer’s instructions. Cytokines were measured and analysed on a Bio-Plex 200 system running the software Bio-Plex Manager 6.4 (Bio-Rad Laboratories). Seventy-five particles per cytokine species were collected and analysed. The standard curves were calculated using five-parameter logistic regression.

### Albumin-binding capacity

To address available site II–specific binding capacity of albumin before and after each ECAD cycle, albumin-binding capacity (ABiC) was measured in plasma samples as previously described [18].

### Quantification of total bile acids

Serum total bile acid (TBA) levels were analysed using an enzymatic colorimetric bile acid assay kit in accordance with the manufacturer’s protocol (Abbott Laboratories, Abbott Park, IL, USA). The analytical measurement range was 1.0–200 μmol/L. Samples with TBA values exceeding 200 μmol/L were diluted manually (1:10) using 0.90 % NaCl.

### Statistical analysis

The prospective study was designed as a non-inferiority replicated crossover trial with up to four replications where an inferiority of at least 10 % was regarded as clinically relevant. The sample size calculations were based on the data of 50 patients of the retrospective pilot study [9]. The data suggested a log-normal distribution for the bilirubin concentrations, an expected ratio of 1 for the means of the two treatments and an intra-subject coefficient of variation of 22.5 %. Furthermore, we requested a type I error of 5 %, leading to 70 cycles of MARS and SPAD to achieve a power of 80 %, including a 10 % dropout rate. The sample size calculations and the statistical analysis by means of linear mixed-effect models were performed with R statistical software [19]. We applied the R package bear [20] for the sample size calculations and R packages lme4 [21] and lmerTest [22] for the statistical analysis. Changes in laboratory and clinical parameters before and after treatment were compared using the Wilcoxon signed-rank test. Data are expressed as median (25th and 75th percentiles) or frequencies, if not otherwise specified.

## Results

### Study population

**Fig. 1:**
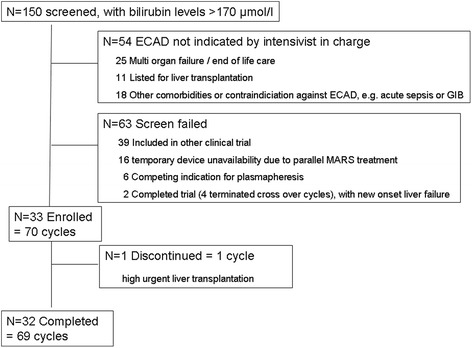
Flowchart of participants within the study period. *ECAD* extracorporeal albumin dialysis, *GIB* gastrointestinal bleeding, *MARS* Molecular Adsorbents Recirculating System

Between 15 August 2010 and 23 February 2013, 33 patients were enrolled in this clinical study. While in 54 patients ECAD was not indicated and/or initiated by the intensivist in charge, screening failed in 117 patients predominantly because of inclusion in other interventional clinical trials (see Fig. 1). One patient underwent a highly urgent liver transplant after randomisation and failed to complete the crossover ECAD cycle and thus was excluded from analysis. Demographic data and baseline characteristics of the study population are summarized in Table 1. According to our SOP, all patients fulfilled the aforementioned ECAD criteria before randomisation for each cycle: PDR_ICG_ 3.9 %/minute (2.58–4.98), total bilirubin 334 μmol/L (263.0–397.0), INR 1.6 (1.30–2.05) and HESA grade III (1.0–4.0).

**Table 1:** Patient characteristics at study inclusion

Characteristics	Data
Age, yr	
Median (IQR)	56 [50–64]
Minimum/maximum	25/75
Male sex, *n* (%)	18 (56.3)
Outcome, *n* (%)	
ICU survival	12 (37.5)
Hospital survival	11 (34.4)
Scores at study inclusion, mean ± SD	
APACHE II	21 ± 6.8
SAPS II	54 ± 16.6
SOFA	13 ± 4.4
Renal function on study inclusion, *n* (%)	
AKIN stage	
1	3 (9.4)
2	11 (34.4)
3	18 (56.3)
Haemodialysis before study inclusion	19 (59.4)
Liver failure, *n* (%)	
Acute-on-chronic	18 (56.3)
Acute	9 (28.1)
Liver graft failure	5 (15.6)

*ICU* intensive care unit, *APACHE II* Acute Physiology and Chronic Health Evaluation II, *SAPS II* Simplified Acute Physiology Score II, *SOFA* Sequential Organ Failure Assessment, *AKIN* Acute Kidney Injury Network, *SD* standard deviation, *IQR* interquartile range

### Performance of ECAD

In total, 69 crossover cycles of ECAD were performed, each consisting of 1 MARS and 1 SPAD treatment. The median durations were 8:00 h (7:55–08:22) for MARS vs. 7:15 h (7:00–7:30) for SPAD (*p* = 0.001). Blood anti-coagulation was maintained mainly with regional citrate infusion (*n* = 52 cycles in MARS vs. *n* = 54 cycles in SPAD; *p* = 0.414), while unfractionated heparin was a minor choice (*n* = 17 in MARS vs. *n* = 15 in SPAD) for blood anti-coagulation. Median application of ECAD was 2 (1–4) cycles, with a minimum of 1 and a maximum of 4 cycles per patient as outlined in the study protocol.

### Effect of MARS and SPAD on paraclinical parameters

#### Liver laboratory parameters

**Fig. 2:**
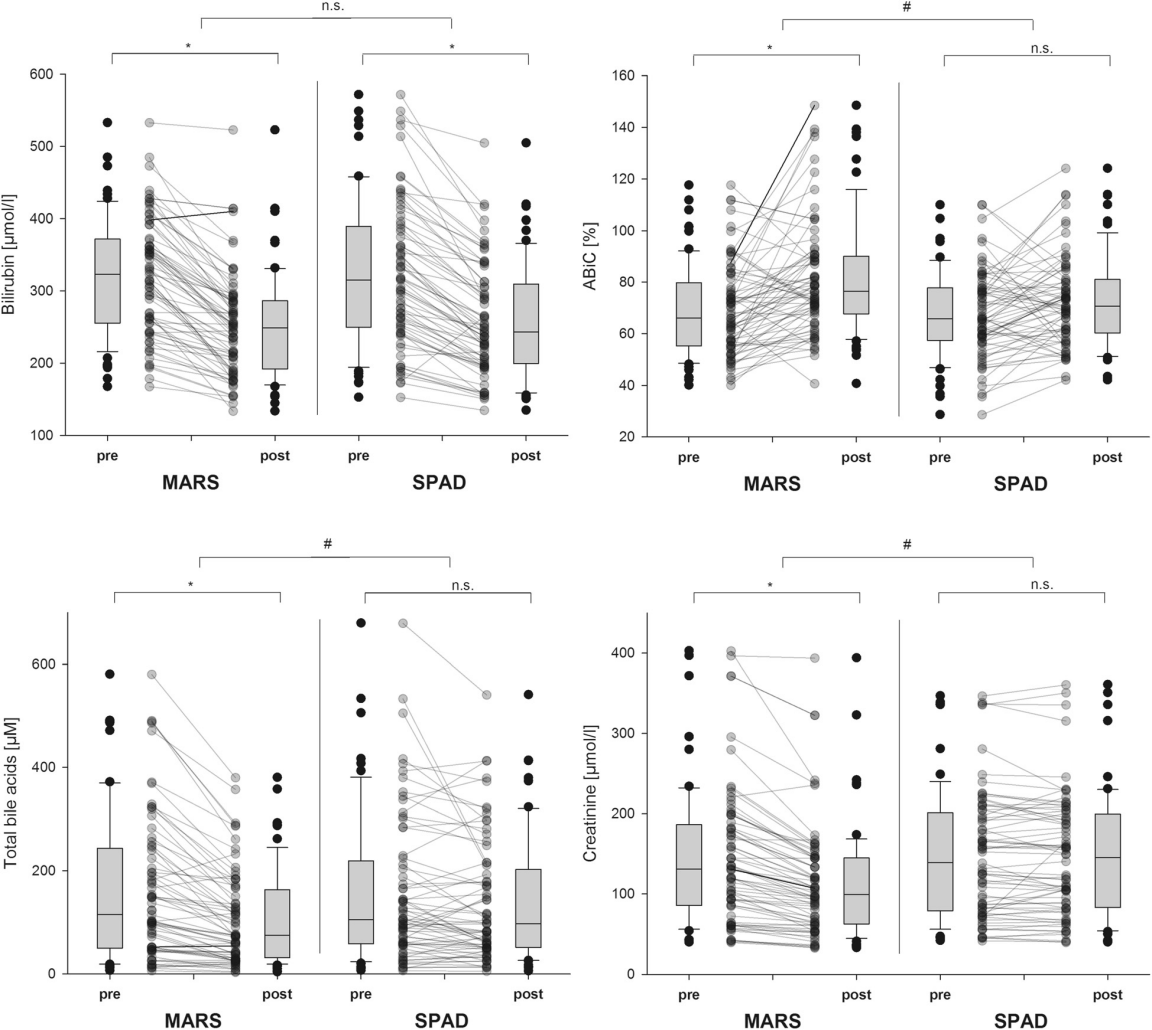
Changes in laboratory parameters representing the primary and secondary study endpoints. Total bilirubin levels, albumin-binding capacity (ABiC), total bile acid concentrations and creatinine levels during albumin dialysis, separated by the applied extracorporeal albumin dialysis (ECAD) systems Molecular Adsorbents Recirculating System (MARS) or single-pass albumin dialysis (SPAD). Box plots represent overall values including all 69 performed ECAD cycles, while bounded dots mark changes of each individual ECAD application. *p < 0.05 significant changes within ECAD; ^#^ *p* < 0.05 significant differences between both devices. *n.s.* non-significant difference

Both systems led to a significant reduction of total plasma bilirubin levels without significant differences between the two devices (*p* = 0.3). Moreover, we could not find any period (*p* = 0.5) or carryover effects (*p* = 0.1) regarding the bilirubin reduction. TBA concentrations were significantly reduced by MARS application, while SPAD resulted in a non-significant reduction (see Table 2 and Fig. 2). After MARS, levels of γ-glutamyl transferase (GGT) were significantly reduced; after SPAD, levels of GGT remained unchanged. Reduction of GGT was significantly different between the devices (*p* = 0.019) (see Table 2). All other investigated paraclinical liver enzymes (alanine aminotransferase, aspartate aminotransferase, alkaline phosphatase, glutamate dehydrogenase, cholinesterase, ammonia and coagulation factor V) demonstrated either marginal or no changes during ECAD application and thus revealed no significant differences between the two devices. These data are provided in Additional file 1: Table S1.

**Table 2:** Liver laboratory parameters, cytokines and kidney retention parameters before and after ECAD

	MARS, median (IQR)			SPAD, median (IQR)		
	Before	After	Difference	Before	After	Difference
Bilirubin, μmol/L	323 (255.5–372.0)	249 (192.0–286.5)	*	315 (249.5–389.5)	243 (199.5–309.5)	*
GGT, μmol/L	1.69 (0.805–4.567)	1.52 (0.760–4.125)	*	1.66 (0.760–3.960)	1.53 (0.795–4.020)	**
TBA, μmol/L	119 (51.6–245.1)	75 (30.9–167.0)	*	105 (58.1–218.9)	97 (50.7–202.4)	**
ABiC, %	67 (55.2–80.6)	76 (67.5–90.4)	*	66 (57.4–77.8)	71 (60.3–81.1)	**
IL-6, pg/ml	142 (62.4–358.9)	123 (63.0–268.8)	n.s.	185 (94.3–369.8)	173 (55.9–385.8)	n.s.
IL-8, pg/ml	66 (46.2–100.9)	65 (49.7–94.9)	n.s.	70 (46.6–124.5)	68 (45.6–108.1)	n.s.
Creatinine, μmol/L	132 (85.5–186.5)	101 (64.0–145.9)	*	139 (79.0–201.5)	146 (83.0–199.8)	**
Urea, mmol/L	11.6 (7.13–17.10)	10.5 (6.60–15.30)	*	12.8 (8.35–18.10)	13.4 (8.93–16.88)	**

*ECAD* extracorporeal albumin dialysis, *MARS* Molecular Adsorbents Recirculating System, *SPAD* single-pass albumin dialysis, *GGT* γ-glutamyl transferase, *TBA* total bile acid, *ABiC* albumin-binding capacity, *IQR* interquartile range, *IL* interleukin, *n.s.* non-significant

*p < 0.05 significant changes within ECAD

**p < 0.05 significant differences between both devices

#### Impact on surrogates for kidney function

The majority (91 %) of patients displayed moderate to severe renal dysfunction according to the Acute Kidney Injury Network (AKIN) criteria [23], and 60 % were on haemodialysis before study inclusion (see Table 1). As markers of residual renal function, median urine output before study inclusion was 477 ml/24 h (63.8–1042.5], accompanied by elevated levels of creatinine (170 μmol/L [112.5–223.5]) and urea (16.3 mmol/L [9.80–21.38]). On the day albumin dialyses were performed, 24-h urine volumes were comparable between both devices (MARS: 278 ml/24 h, 25.0–732.5; SPAD: 300 ml/24 h, 21.0–988.0). Regarding dialysis capacity, MARS resulted in significant reduction of plasma creatinine levels, while SPAD did not significantly reduce plasma creatinine (see Table 2 and Fig. 2). For plasma urea levels, the same pattern was observed. MARS led to a higher reduction in kidney retention parameters than SPAD (*p* < 0.001 for creatinine and *p* < 0.001 for urea, respectively).

#### Albumin-binding capacity

ABiC, as a marker of functional status of the albumin molecule, significantly increased during MARS administration, but it remained unchanged in SPAD application. Changes of ABiC values were significantly different between the systems (*p* = 0.025), whereas human serum albumin levels did not change during treatment (see also Fig. 2 and Table 2).

#### Cytokine removal

To evaluate cytokine removal by ECAD, various human cytokine levels were determined before and after each cycle. However, only human IL-6 and IL-8 levels were frequently elevated. Neither MARS nor SPAD resulted in significant cytokine reduction, and there was no difference between the two ECAD devices (Table 2 and Additional file 1: Figure S1). Moreover, neither the citrate nor the heparin anti-coagulation strategy had an impact on IL-6 or IL-8 removal (Additional file 1: Table S2).

#### Acid–base status and electrolytes

**Fig. 3:**
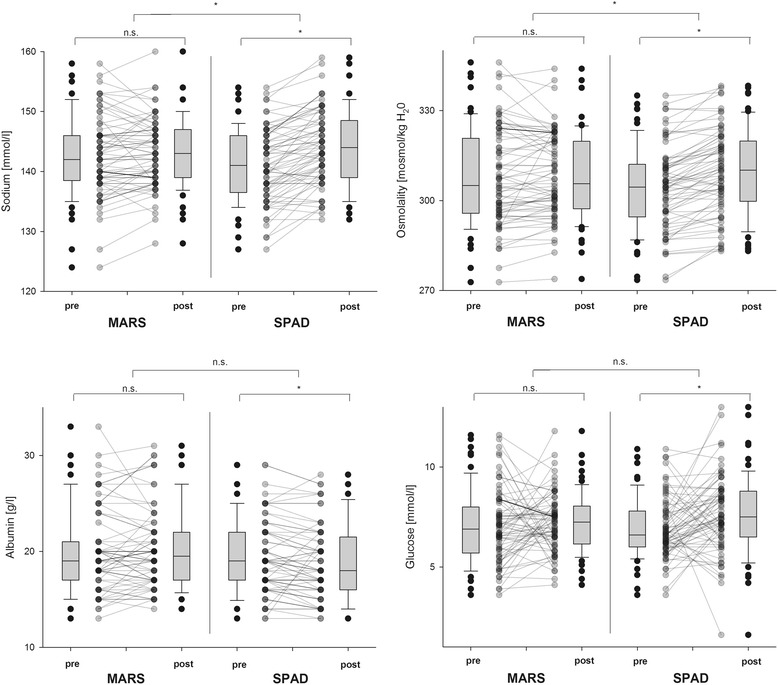
Changes in laboratory markers hinting to metabolic disturbances and electrolyte derangements in both extracorporeal albumin dialysis (ECAD) devices. Box plots represent overall values including all 69 performed ECAD cycles, and bounded dots mark changes of each individual ECAD application. *MARS* Molecular Adsorbents Recirculating System, *SPAD* single-pass albumin dialysis, *n.s.* non-significant difference

Median pH values significantly increased during SPAD application (*p* < 0.001). Increased levels of standard bicarbonate (SBC) and base excess (BE) pointed to a non-respiratory cause of pH increase during the SPAD procedure. MARS led to only non-significant changes in pH, SBC and BE levels. Lactate levels increased during the application of both devices. While MARS led to an increase of 0.3 mmol/L (−0.18 to +0.58), lactate levels increased during SPAD by 0.6 mmol/L (0.05–1.00). Regarding parameters of acid–base status, only lactate levels showed significant differences between the devices (*p* = 0.041). During the use of both devices, median calcium (MARS: *p* = 0.002; SPAD: *p* = 0.001) and potassium levels (MARS: *p* = 0.027; SPAD: *p* = 0.005) significantly decreased, while sodium (*p* < 0.001) and glucose (*p* = 0.003) levels showed an increase only during SPAD. The differences in calcium and sodium levels were significantly different when both devices were compared (see Table 3 and Fig. 3). Using the equation published by Fazekas et al. [24], we found that osmolality significantly differed when we compared both devices (*p* < 0.001). While SPAD led to an increase (*p* < 0.001), osmolality remained unchanged during MARS (*p* = 0.252) (Table 3 and Fig. 3). Separating anti-coagulation strategies (heparin vs. citrate) in both systems, citrate application during SPAD led to significant metabolic changes towards non-respiratory alkalosis (indicated by pH, BE and SBC increase) accompanied by an increase of lactate and a decrease in systemic ionized calcium levels. Moreover, sodium levels and osmolality also increased during SPAD using citrate anti-coagulation. In contrast, neither heparin application in either device nor citrate infusion during MARS led to similar disturbances in acid–base states, electrolyte balance or osmolality (see Additional file 1: Table S2).

**Table 3:** Acid–base parameters and electrolytes before and after ECAD

	MARS, median (IQR)			SPAD, median (IQR)		
	Pre-ECAD	Post-ECAD	Difference	Pre-ECAD	Post-ECAD	Difference
pH	7.42 (7.374–7.447)	7.42 (7.390–7.449)	n.s.	7.40 (7.356–7.424)	7.43 (7.386–7.454)	*
SBC, mmol/L	27.3 (24.03–31.48)	27.5 (24.70–30.60)	n.s.	25.1 (22.75–27.50)	27.8 (24.45–30.60)	*
Base excess, mmol/L	3.9 (0.03–8.35)	3.8 (0.70–7.20)	n.s.	1.3 (−1.40/+3.85)	4.0 (0.40/7.65)	*
Lactate, mmol/L	1.9 (1.30–2.70)	2.1 (1.70–3.00)	*	1.8 (1.30–2.75)	2.5 (1.70–3.60)	*^,^**
Sodium, mmol/L	142 (138.3–146.0)	143 (139.0–147.0)	n.s.	141 (136.5–146.0)	144 (139.0–148.5)	*^,^**
Potassium, mmol/L	4.5 (4.20–4.70)	4.2 (3.90–4.60)	*	4.4 (4.15–4.70)	4.2 (3.90–4.60)	*
Calcium, mmol/L	1.24 (1.140–1.330)	1.15 (1.090–1.260)	*	1.21 (1.115–1.310)	1.06 (0.890–1.145)	*^,^**
Glucose, mmol/L	6.9 (5.70–8.00)	7.3 (6.15–8.05)	n.s.	6.6 (6.00–7.80)	7.5 (6.50–8.80)	*
Osmolality, mOsmol/kg H_2_O	305.1 (295.85–320.79)	305.7 (297.24–319.79)	n.s.	304.5 (294.53–312.01)	310.1 (299.76–319.85)	*^,^**

*ECAD* extracorporeal albumin dialysis, *MARS* Molecular Adsorbents Recirculating System, *SPAD* single-pass albumin dialysis, *SBC* standard bicarbonate, *IQR* interquartile range, *n.s.* non-significant

*p < 0.05 significant changes within ECAD

**p < 0.05 significant differences between both devices

#### Haemoglobin and blood coagulation

Levels of haemoglobin (MARS: *p* = 0.002; SPAD: *p* = 0.005), haematocrit (MARS: *p* = 0.005; SPAD: *p* = 0.016) and INR (MARS: *p* = 0.449; SPAD: *p* = 0.049) changed only slightly during ECAD, showing no significant differences between the devices. However, platelets significantly decreased during MARS (−8, −18.9 to +1.5; *p* < 0.001), but not during SPAD application (−2, −11.5 to +9.0; *p* = 0.286) and thus were significantly different between both devices (*p* = 0.032) (see Additional file 1: Table S3).

### Effect of MARS and SPAD on clinical parameters

#### Hepatic encephalopathy and neurological scoring

HE was analysed using the HESA score, consciousness was evaluated by Glasgow Coma Scale score and grade of sedation was assessed using the Ramsay score. All of these parameters were similar between the devices and showed no significant changes during the administration of MARS or SPAD. Moreover, separating ECAD cycles under sedative medication (*n* = 20) from cycles without sedative medication (*n* = 49) revealed no changes in any of the aforementioned scores after completion of one crossover cycle (MARS and SPAD or vice versa) (for convenience, see Additional file 1: Table S4).

#### Haemodynamic parameters

Mean arterial pressure increased during ECAD application, but only in SPAD did it reach statistical significance. However, we found no significant difference when we compared both ECAD devices. All other values in the haemodynamic profile (i.e., heart rate, CVP, central venous oxygen saturation and dosage of vasopressor support) were similar before and after each ECAD cycle and thus also showed no significant differences between the devices. In 13 MARS cycles and 15 SPAD cycles, patients had intensified haemodynamic monitoring using transpulmonary thermodilution. The values for cardiac index, ELWI and ITBI were comparable before and after each ECAD cycle as well as between the devices (see Additional file 1: Table S5).

#### Thermal balance

Evaluation of thermal balance during application of both devices revealed slightly increasing body temperature under MARS application (+0.3 °C, −0.15 to +0.75) compared with SPAD treatment (−0.1 °C, −0.55 to +0.35).

#### Transfusion rates during ECAD

The median transfusion rates were 1 (0–1) for erythrocytes, 0 (0–0) for fresh frozen plasma and 0 (0–0) for platelets, without differences between the devices.

## Discussion

Following the autointoxication hypothesis of liver failure [1], the aim of using liver support systems is to reduce endogenous liver toxins. They carry the potential to reduce not only water-soluble toxic substances (e.g., by means of conventional haemodialysis) but also hydrophobic agents (e.g., by using albumin as a dialysis solution). Due to its biochemical structure, albumin is able to bind numerous endo- and exogenous substrates, such as bilirubin and bile acids [25], that can thus be cleared via ECAD (i.e., MARS or SPAD).

In this prospective crossover study, we compared these two extracorporeal liver support systems with a particular focus on their effect on bilirubin levels and further clinical and laboratory parameters. We found equivalent significant reductions in plasma bilirubin levels in both MARS and 4 % SPAD, without differences between the methods. Measurement of bilirubin, both unconjugated and conjugated, is common in intensive care units to detect serious disorders (e.g., cholestasis or haemolysis). Bilirubin levels were found to be associated with prognosis in the critical care setting [26] and particularly in liver failure. With respect to extracorporeal liver support therapy, bilirubin is often used as one of several parameters to indicate therapy and, in addition, to assess effective elimination of albumin-bound substances. Bilirubin can easily pass the haemodialysis filter; however, due to its lipophilic structure and its low water solubility, a carrier is necessary to eliminate bilirubin via haemodialysis. Human serum albumin, with its three bilirubin binding sites [27], has a high capacity to remove bilirubin during albumin dialysis. In a retrospective analysis, we had found similar reduction rates of bilirubin in patients treated with either MARS or SPAD [9]. We used these results for a sample size calculation and bilirubin thereby became the primary endpoint in our present study.

Although not routinely assessed, other parameters, such as bile acids or ABiC, might be of superior clinical significance in defining excretory liver dysfunction compared with bilirubin levels. For example, toxic bile acids can induce hepatocyte and biliary epithelial cell necrosis [28] and are therefore of potential prognostic significance [29, 30]. Furthermore, they might contribute to remote organ dysfunction such as cirrhotic cardiomyopathy [31] or reduced systemic vascular resistance [32]. In contrast to bilirubin levels, TBA concentrations were significantly reduced only during MARS and not during SPAD treatment. MARS was previously shown to reduce bile acid concentrations in vivo [33–35]. In the case of SPAD, two in vitro studies [8, 36] and one small uncontrolled study [7] addressing the clearance of bile acids have been published. Interestingly, in the study of Sauer et al. [8], MARS and SPAD were comparable in bile acid clearance; in the study of Benyoub et al. [7], bile acids were significantly removed during a 10-h SPAD (3.2 % albumin concentration) procedure with a dialysate flow rate of 1000 ml/h. These results are in clear contrast to our findings, the latter possibly being due to higher dialysate flow rate and longer treatment period, resulting in a much higher dialysis dose. One potential explanation for the discrepancy in the in vitro studies could be the different dialysis modes used (continuous venovenous haemodiafiltration in vitro vs. continuous venovenous haemodialysis [CVVHD] in our study). However, with a molecular weight of about 500 Da, bile acids should be removed more effectively in the dialysis mode; indeed, Benyoub et al. used CVVHD as well [7]. The more likely explanation is an inherent limitation of the in vitro setting representing a one-compartment model (i.e., the intravascular compartment). In vivo elimination of bile acids is complicated by diffusion of bile acids from tissue and by ongoing bile acid production. Differences in the effectiveness of MARS and SPAD in removing bile acids could thereby be unmasked.

With respect to potential toxicity of bile acids and the higher removal rate of bile acids during MARS treatment in our study, the reduction of GGT levels during MARS could suggest bile duct regeneration, as high levels of GGT correlate to bile duct lesions [37]. Thus, it can be speculated that reduction and/or expression pattern change of bile acids (i.e., allocation of conjugated to unconjugated) could improve the disease course. However, recent large randomised clinical trials of extracorporeal liver support, in either acute or acute-on-chronic liver failure [2, 4], did not focus on bile acid removal, so further study would be needed to directly address this possibility.

Human serum albumin is the major carrier protein for a multitude of endogenous and exogenous substances. Many accumulating substances in liver failure are transported bound to albumin. ABiC was developed to assess the binding site II–specific binding capacity of human serum albumin, the binding site of, for example, bile acids [38]. ABiC correlated inversely with severity and 30-day mortality in patients with decompensated cirrhosis [18]. ABiC was exclusively increased during MARS treatment in the present study. Such an improvement of ABiC in MARS treatment was demonstrated previously [39]. In the present study, SPAD was not able to improve ABiC. These findings might be at least in part an accompanying effect of the results seen in bile acids, as higher bile acid levels with higher binding at binding site II might lead to reduced ABiC. Another potential explanation for this finding may be in the stabilizers added to pharmaceutical albumin preparations. These stabilizers may occupy binding sites of albumin (e.g., octanoate, which also binds to albumin binding site II). On the one hand, these stabilizers can be bound to the adsorption columns in the albumin circuit in MARS, thereby improving capacity to remove albumin-bound substances such as bile acids from the blood circuit. On the other hand, the demonstrated higher increase of octanoate concentrations in blood of patients during SPAD treatment may contribute to the less increased ABiC in comparison to MARS treatment [11]. Of note, the albumin solution used in the present study contained the stabilizer octanoate equivalent to caprylate. Whether removing these stabilizers (e.g., by charcoal filters) before albumin dialysis might overcome these shortcomings in SPAD therapy remains elusive.

As acute or chronic renal insufficiency displays a common complication in liver dysfunction, removal of water-soluble substances, such as urea and creatinine, is often required during ECAD. However, assessment of renal dysfunction by traditional scoring systems (i.e., AKIN criteria) is not always applicable [40], and some patients require haemodialysis for reasons not related to creatinine levels and urine output (e.g., hypervolemia or acidosis). In the present study, the majority of the critically ill patient cohort required renal replacement therapy before study inclusion. Thus, ECAD treatment was coupled to conventional haemodialysis circuits. The ability to decrease water-soluble substances in our study was higher in MARS than in SPAD treatment, most due to differing dialysate flow rates among the ECAD devices (MARS 2000 ml/h vs. SPAD 700 ml/h). In the clinical setting, this might be of minor relevance, as it could easily be compensated for if a 7-h SPAD treatment were followed by conventional continuous renal replacement therapy using the same dialysing machine with conventional dialysing solution and a higher dialysate flow rate. However, it must be noted that variances in the reduction of water-soluble substances may also be altered by residual endogenous renal function, possibly influencing the efficacy of ECAD therapy. Nevertheless, due to the crossover study design, the influence of either MARS or SPAD on alteration of kidney retention parameters in the present study may overcome these shortcomings.

Pro-inflammatory cytokines are suspected to induce and/or augment hepatocellular damage and cholestasis, thereby contributing to the course of liver failure [41]. However, there is evidence that growth factors and pro-inflammatory cytokines are involved in liver regeneration and proliferation of hepatocytes. Data regarding the influence of ECAD on systemic cytokine levels in patients with acute or acute-on-chronic liver failure are conflicting [42–44]. Removal of cytokines via albumin dialysis has been demonstrated. In addition, circulating cytokine levels could be affected by either removal by dialysis or induced changes in the rate of production. Moreover, different anti-coagulation strategies could possibly alter cytokine removal. Thus, elevated IL-8 levels were shown to be removed via CVVHD under regional citrate anti-coagulation, but not using heparin anti-coagulation. Interestingly, IL-6 levels were altered by neither citrate nor heparin anti-coagulation [45]. However, researchers in other clinical studies could not find any differences regarding cytokine removal during conventional haemodialysis using different anti-coagulation strategies [46, 47]. In this respect, combining albumin dialysis with a cytokine adsorption filter may be superior regarding cytokine removal [48]. However, the clinical relevance of elevated cytokine levels in patients with liver failure is not fully understood, as cytokines could enhance cell damage as well as induce liver regeneration [43, 49, 50]. In our study, elevated systemic cytokine levels were lowered neither during MARS nor during SPAD application. Moreover, neither citrate nor heparin anti-coagulation altered cytokine levels in the present study.

Both systems were safe in providing extracorporeal liver support in critically ill patients, particularly in view of bleeding complications, transfusion rates and haemodynamic stability. Clinically non-significant changes in thermal balance may be explained by more pronounced warming of the dialysate solution during MARS (two heating devices: dialysis machine and MARS monitor) compared with SPAD (one single heating during haemodialysis). In SPAD treatment, we found higher rates of metabolic derangement (increase in pH, BE and lactate values) and electrolyte disturbances (decreasing calcium levels and increasing sodium levels), resulting in osmolality displacements. These changes were limited to patients receiving citrate anti-coagulation, hinting at a relative overdosing of citrate [51], most likely due to the low dialysate flow rate of 700 ml/h in SPAD treatment and the use of commercially available citrate solutions designed for conventional haemodialysis. To compensate for this apparent disadvantage (1) dialysate flow rates could be increased or (2) lower concentrated sodium citrate solutions during SPAD procedures running on regional citrate anti-coagulation could be used. On one hand, increasing the dialysate flow rate would reduce the treatment duration or it must be accounted for by reducing the albumin content. Increasing the albumin concentration as well as the dialysate flow rate, on the other hand, would enhance costing of SPAD, abolishing the savings of around €1500 per treatment cycle using the described setting of our study in comparison to MARS (only material costing, not including personnel for device build-up).

Regarding HE, patients scored a median of grade III on the HESA scale of four grades and 14 on the Glasgow Coma Scale, indicating presence of HE in the patient cohort. Albumin dialysis was not able to significantly affect HE grade among the study population, although this was shown before by Hassanein et al. [3]. Competitive sedative medication allowing invasive mechanical ventilation in this critically ill patient cohort may represent one explanation for this negative finding. Moreover, the study was not designed to improve HE. We evaluated HE scores after each ECAD cycle, which was not expected to provide enough detoxification capacity to alter the HE course. This is consistent with the findings of the study of Hassanein et al., in which most patients needed at least two treatments before HE improved.

Two limitations of our study are its single-centre design and the relatively small number of patients. However, we made a careful sample size calculation based on retrospective data from our institution. As liver failure is a rare disease and patients who require extracorporeal liver support must be selected carefully, the number of potential study patients is limited. Therefore, we implemented the possibility for multiple applications of up to four crossover cycles of albumin dialysis per patient in the study design. This was accounted for in the sample size analysis. Moreover, the crossover study design revealed some major advantages in the present analysis: (1) there are no confounding factors with respect to comparability between the control- and test-group, as both were represented by the same patient/cycle, and (2) requirements of type I or type II errors are generally comparable in cross-crossover design studies compared with parallel group trials, at a lower sample size [52]. In addition, we assessed only a limited number of potential outcome-relevant factors (i.e. cytokine, bile acid or bilirubin levels and ABiC). We cannot exclude that there are significant differences regarding further parameters potentially influenced by ECAD, such as plasma levels of other toxic substances or other albumin-related factors (e.g., heavy metal binding capacity). Furthermore, the detoxification efficiency of the closed albumin circuit (MARS) and the open albumin-enriched dialysis system (SPAD) are dependent on some confounding factors; for example, high solute concentrations of albumin-bound substances might result in early saturation of adsorbers in MARS, while low concentrations might leave residual adsorption capacity. However, in SPAD, higher solute concentrations potentially will not lead to saturation of the dialysis process. In addition, longer treatment times or higher dialysate flow rates in principle could result in higher clearance of substances, albeit accompanied by higher treatment costs. The decision to run MARS in the described setting was based on the manufacturer’s recommendation for duration of MARS [1]. This approach was previously used in larger multicentre trials on acute and acute-on-chronic liver failure, respectively [2, 4]. Treatment settings of SPAD were based on a previous in vitro study and on our own retrospective in vivo analysis [8, 9]. To preclude further confounders, blood flow rates were equal during respective MARS and SPAD treatments. Moreover, the crossover design itself limits bias in comparing an open against a closed system, as all patients receive both treatments.

## Conclusions

This prospective, randomised, controlled crossover study demonstrated the investigated albumin dialysis procedures to be safe for temporary extracorporeal liver support. Overall, both devices displayed comparable results for most clinical and paraclinical parameters in our critically ill patient cohort, especially in light of bilirubin reduction. However, particular focus should be placed on metabolic derangements and electrolyte disturbances in case of regional citrate anti-coagulation and reduced renal haemodialysis efficacy (i.e., creatinine and urea levels) caused by reduced dialysis flow rates during SPAD application. Increasing dialysis efficacy of SPAD by increasing dialysate flow rate and extending treatment duration might compensate for this disadvantage. However, this needs to be investigated further. In addition, it would increase treatment costs of SPAD, thereby decreasing the cost savings in comparison to MARS. Moreover, in view of recent aspects regarding the pathophysiology of liver failure and consecutive remote organ failure, it remains speculative if MARS may provide advantages over SPAD by reducing bile acid concentration and improving ABiC. Thus, further studies addressing the timing and duration of ECAD, particularly in view of altering clinical and paraclinical parameters beyond bilirubin reduction (e.g., HE, cytokine removal, ABiC enhancement and bile acid changes), are warranted. Furthermore, meaningful clinical outcomes, such as patient survival, time on mechanical ventilation, vasopressor support or course of HE, should be addressed.

## Key messages

- This prospective crossover study shows that both albumin dialysis devices, MARS and SPAD, were able to reduce patients’ bilirubin levels to the same amount (primary study endpoint).
- Both investigated albumin dialysis procedures were safe for temporary extracorporeal liver support.
- However, in view of recent aspects regarding the pathophysiology of liver failure and consecutive remote organ failure, MARS provided advantages in reducing bile acid concentration and improving ABiC.
- Particular focus should be placed on the reduced renal haemodialysis efficacy for creatinine and urea levels during SPAD application.
- Application of SPAD using conventional regional citrate anti-coagulation should be performed with caution and efficacy of treatment need to be monitored more carefully, especially in light of metabolic derangements, electrolyte balance and osmolality.

## Additional file

Additional file 1: Table S1. Course and changes of liver enzymes before and after ECAD. Significant changes (p<0.05) within ECAD are marked with *. Significant differences between both devices (p<0.05) were marked with #. **Table S2:** Course and changes of acid base parameters and electrolytes before and after ECAD. Significant changes (p<0.05) within ECAD are marked with *. Significant differences between both devices (p<0.05) were marked with #. **Table S3:** Course and changes of blood and coagulation factors before and after ECAD. Significant changes (p<0.05) within ECAD are marked with *. Significant differences between both devices (p<0.05) were marked with #. **Table S4:** Course and changes of neurological parameters before and after ECAD. Significant changes (p<0.05) within ECAD are marked with *. Significant differences between both devices (p<0.05) were marked with #. **Table S5:** Course and changes of hemodynamic parameters before and after ECAD. Significant changes (p<0.05) within ECAD are marked with *. Significant differences between both devices (p<0.05) were marked with #. **Figure S1:** Changes in Cytokine levels, separated to the applied ECAD systems. Box plots represent overall values including all 69 performed ECAD cycles, while bounded dots mark changes of each individual ECAD application. (MARS: Molecular adsorbent recirculating system; SPAD: Single pass albumin dialysis; n.s.: non-significant difference. (DOC 458 kb)

## Abbreviations

ABiC: albumin-binding capacity
AKIN: Acute Kidney Injury Network
APACHE II: Acute Physiology and Chronic Health Evaluation II
BE: base excess
CVP: central venous pressure
CVVHD: continuous venovenous haemodialysis
ECAD: extracorporeal albumin dialysis
ELWI: extravascular lung water index
GGT: γ-glutamyl transferase
GIB: gastrointestinal bleeding
HE: hepatic encephalopathy
HESA: Hepatic Encephalopathy Scoring Algorithm
ICU: intensive care unit
IL: interleukin
INR: international normalised ratio
IQR: interquartile range
ITBI: intrathoracic blood volume index
MARS: Molecular Adsorbents Recirculating System
PDR_ICG_: plasma disappearance rate of indocyanine green
SAPS II: Simplified Acute Physiology Score II
SBC: standard bicarbonate
SOFA: Sequential Organ Failure Assessment
SOP: standard operating procedure
SPAD: single-pass albumin dialysis
TBA: total bile acid
