# Supplementary material for: Molecular adsorbent recirculating system and single-pass albumin dialysis in liver failure – a prospective, randomised crossover study
Source: Crit Care. 2016 Jan 4;20:2. doi: 10.1186/s13054-015-1159-3 (PMC4699252; doi:10.1186/s13054-015-1159-3)

**Molecular adsorbent recirculating system and single pass albumin dialysis in liver failure – a prospective randomized cross-over study**

**- Supplemental Material -**

Christoph Sponholz, Katja Matthes, Dina Rupp, Wolf Backaus, Sebastian Klammt, Diana Karailieva, Astrid Bauschke, Utz Settmacher, Matthias Kohl, Mark G. Clemens, Steffen Mitzner, Michael Bauer and Andreas Kortgen

**Corresponding author:**

Andreas Kortgen

Department of Anaesthesiology and Critical Care Medicine

Jena University Hospital

Erlanger Allee 101

D – 07747 Jena

Germany

Phone: +49-3641-9323184

Fax: +49-3641-9323112

Mail: andreas.kortgen @med.uni-jena.de

Table S1:

Course and changes of liver enzymes before and after ECAD. Significant changes (p<0.05) within ECAD are marked with *. Significant differences between both devices (p<0.05) were marked with ^#^.

|  |  | MARS | | | | | | SPAD | | | | | |
| --- | --- | --- | --- | --- | --- | --- | --- | --- | --- | --- | --- | --- | --- |
|  |  | pre | | post | | difference | | pre | | post | | difference | |
| ASAT | [µmol/l] | 1.32 | [0.855 / 2.542] | 1.36 | [0.850 / 2.350] | -0.09 | [-0.213 / +0.020] | 1.42 | [0.845 / 2.585] | 1.53 | [0.840 / 2.420] | -0.01 | [-0.135 / +0.085] |
| ALAT | [µmol/l] | 0.80 | [0.460 / 1.617] | 0.74 | [0.470 / 1.700] | -0.01 | [-0.078 / +0.030] | 0.75 | [0.470 / 1.465] | 0.81 | [0.505 / 1.575] | 0.00 | [-0.040 / +0.040] |
| GLDH | [µmol/l] | 86 | [50.0 / 223.0] | 82 | [50.0 / 219.3] | -4 | [-47.5 / 0.00) | 73 | [50 / 297.5] | 84 | [50.0 / 266.5] | 0 | [-26.0 / +1.0] |
| AP | [µmol/l] | 2.40 | [1.590 / 4.170] | 2.15 | [1.555 / 3.415] | -0.13 | [-0.425 / +0.060] | 2.14 | [1.502 / 4.900] | 2.21 | [1.553 / 4.715] | -0.05 | [-0.260 / +0.110] |
| CHE | [µmol/l] | 21 | [15.0 / 28.0] | 20 | [14.0 / 28.0] | -1 | [-3.0 / 0.0] | 21 | [15.0 / 29.0] | 19 | [15.0 / 29.0] | 0 | [-26.0 / +1.0] |
| Ammonia | [µmol/l] | 34 | [23.8 / 48.3] | 33 | [24.0 / 46.0] | 0 | [-5.8 / +4.8] | 37 | [29.3 / 44.0] | 34 | [25.0 / 48.0] | -2 | [-6.0 / +7.0] |
| Factor-V | [%] | 63 | [33.5 / 113.0] | 64 | [30.3 / 107.5] | -3 | [-10.0 / +2.0] | 55 | [33.8 / 106.0] | 56 | [32.0 / 109.0] | -2 | [-7.5 / +2.0] |
| MARS: Molecular adsorbent recirculating system; SPAD: Single pass albumin dialysis  ASAT: Aspartate aminotransferase; ALAT: Alanine aminotransferase; GLDH: Glutamate dehydrogenase; AP: Alkaline phosphatase; CHE: Cholinesterase  values represent median and interquartile range | | | | | | | | | | | | | |

Table S2: Course and changes of acid base parameters and electrolytes before and after ECAD. Significant changes (p<0.05) within ECAD are marked with *. Significant differences between both devices (p<0.05) were marked with ^#^.

| MARS |  | Citrate (n=52) | | | | | | | | | Heparin (n=17) | | | | | | |
| --- | --- | --- | --- | --- | --- | --- | --- | --- | --- | --- | --- | --- | --- | --- | --- | --- | --- |
|  |  | pre | | | post | | | | difference | | pre | | post | | difference | | |
| pH |  | 7.42 | [7.366 / 7.447] | | 7.42 | | [7.391 / 7.450] | | 0.00 | [-0.021 / +0.041] | 7.41 | [7.386 / 7.452] | 7.42 | [7.365 / 7.438] | -0.01 | [-0.031 / +0.020] | |
| SBC | [mmol/l] | 27.7 | [24.18 / 32.03] | | 27.9 | | [25.80 / 32.10] | | -0.3 | [-1.25 / +2.80] | 25.5 | [22.85 / 29.30] | 25.6 | [23.03 / 28.35] | -0.6 | [-1.73 / +0.50] | |
| Base excess | [mmol/l] | +4.3 | [+0.25 / +8.93] | | +4.5 | | [+2.10 / +9.03] | | -0.05 | [-1.58 / +3.05] | +1.1 | [-1.25 / +5.25] | +1.3 | [-1.58 / +4.58] | -1.0 | [-2.05 / +0.55] | |
| Lactate | [mmol/l] | 1.9 | [1.33 / 3.08] | | 2.2 | | [1.90 / 3.20] | | +0.4 | [-0.10 / +0.78] | 1.6 | [1.15 / 2.40] | 1.7 | [1.20 / 2.28] | -0.1 | [-0.40 / +0.28] | |
| Calcium | [mmol/l] | 1.25 | [1.142 / 1.330] | | 1.15 | | [1.085 / 1.275] | | **-0.05*** | [-0.158 / +0.058] | 1.17 | [1.135 / 1.295] | 1.18 | [1.100 / 1.268] | -0.04 | [-0.078 / +0.003] | |
| Sodium | [mmol/l] | 143 | [138.3 / 146.0] | | 144 | | [140.0 / 147.0] | | **+1*** | [-1.0 / +3.0] | 140 | [138.5 / 146.5] | 140 | [137.0 / 145.8] | -1 | [-2.0 / +0.8] | |
| Osmolality | [mosmol/Kg H_2_0] | 306.3 | [296.10 / 321.14] | | 309.9 | | [299.78 / 320.72] | | **+1.6^*^** | [-1.54 / +4.63] | 301.6 | [294.55 / 320.91] | 299.7 | [292.95 / 311.94] | -1.5 | [-5.86 / +0.21] | |
| IL-6 | [pg/ml] | 134 | [56.7 / 367.9] | | 124 | | [57.6 / 342.7] | | +3 | [-82.9 / +44.7] | 167 | [119.6 / 298.6] | 105 | [66.5 / 246.4] | -23 | [-79.0 / +30.2] | |
| IL-8 | [pg/ml] | 60 | [44.1 / 101.8] | | 68 | | [49.4 / 105.1] | | +5 | [-21.2 / +31.4] | 69 | [51.9 / 109.3] | 62 | [50.1 / 85.8] | -7 | [-21.1 / +27.2] | |
|  |  |  |  | |  | |  | |  |  |  |  |  |  |  |  | |
| SPAD |  | Citrate (n=54) | | | | | | | | | Heparin (n=15) | | | | | | |
|  |  | pre | | | | post | | | difference | | pre | | post | | difference | | |
| pH |  | 7.40 | | [7.355 / 7.429] | | 7.44 | | [7.395 / 7.463] | **+0.04*** | [+0.004 / +0.060] | 7.39 | [7.354 / 7.418] | 7.41 | [7.285 / 7.432] | -0.01 | | [-0.048 / +0.014] |
| SBC | [mmol/l] | 25.6 | | [22.83 / 28.15] | | 29.2 | | [26.05 / 31.00] | **+3.1*** | [+1.28 / +5.43] | 24.3 | [22.50 / 27.20] | 23.6 | [20.20 / 25.70] | -1.0 | | [-2.60 / -0.20] |
| Base excess | [mmol/l] | +1.7 | | [-1.43 / +4.73] | | +5.8 | | [+2.10 / +7.90] | **+3.4*** | [+1.20 / +5.98] | -0.3 | [-1.40 / +3.30] | -0.6 | [-4.10 / -1.80] | -1.2 | | [-2.90 / -0.10] |
| Lactate | [mmol/l] | 1.8 | | [1.30 / 2.83] | | 2.8 | | [1.70 / 3.73] | **+0.7*** | [+0.18 / +1.40] | 2.2 | [1.50 / 2.40] | 2.4 | [1.60 / 3.30] | +0.2 | | [-0.10 / +0.40] |
| Calcium | [mmol/l] | 1.22 | | [1.110 / 1.310] | | 1.02 | | [0.825 / 1.100] | **-0.21*** | [-0.313 / -0.085] | 1.21 | [1.170 / 1.330] | 1.15 | [1.090 / 1.220] | **-0.08*** | | [-0.090 / -0.030] |
| Sodium | [mmol/l] | 141 | | [137.0 / 146.0] | | 145 | | [141.0 / 150.0] | **+4^*^** | [+2.8 / +6.3] | 140 | [135.0 / 144.0] | 138 | [135.0 / 143.0] | -1 | | [-2.0 / +0.0] |
| Osmolality | [mosmol/Kg H_2_0] | 305.8 | | [296.38 / 312.91] | | 313.2 | | [304.01 / 321.47] | **+6.1^*^** | [+3.42 / +9.59] | 296.5 | [288.67 / 306.54] | 300.4 | [290.20 / 304.76] | +0.7 | | [-2.08 / +1.56] |
| IL-6 | [pg/ml] | 172 | | [91.7 / 524.9] | | 172 | | [62. 9 / 391.3] | -7 | [-90.2 / +46.6] | 196 | [112.3 / 252.2] | 192 | [52.7 / 374.1] | +22 | | [-44.4 / +145.8] |
| IL-8 | [pg/ml] | 63 | | [41.5 / 131.8] | | 67.5 | | [43.5 / 107.9] | +3 | [-18.1 / +21.6] | 85 | [62.6 / 114.4] | 70 | [59.9 / 113.7] | -1 | | [-27.0 / +22.4] |
| MARS: Molecular adsorbent recirculating system; SPAD: Single pass albumin dialysis  SBC: Standard bicarbonate; IL: Interleukin  values represent median and interquartile range | | | | | | | | | | | | | | | | | |

Table S3: Course and changes of blood and coagulation factors before and after ECAD. Significant changes (p<0.05) within ECAD are marked with *. Significant differences between both devices (p<0.05) were marked with ^#^.

|  |  | MARS | | | | | | SPAD | | | | | |
| --- | --- | --- | --- | --- | --- | --- | --- | --- | --- | --- | --- | --- | --- |
|  |  | pre | | post | | difference | | pre | | post | | difference | |
| Hemoglobin | [mmol/l] | 5.5 | [5.30 / 5.70] | 5.3 | [5.00 / 5.60] | **-0.2*** | [-0.50 / +0.10] | 5.4 | [5.20 / 5.70] | 5.3 | [5.20 / 5.60] | **-0.2*** | [-0.40 / +0.10] |
| Hematocrite |  | 0.26 | [0.250 / 0.270] | 0.25 | [0.240 / 0.270] | **-0.01*** | [-0.020 / +0.010] | 0.26 | [0.250 / 0.270] | 0.25 | [0.240 / 0.270] | **-0.01*** | [-0.020 / +0.010] |
| INR |  | 1.5 | [1.30 / 2.17] | 1.5 | [1.30 / 2.08] | **0.0*** | [-0.10 / + 0.10] | 1.6 | [1.30 / 2.00] | 1.7 | [1.30 / 2.15] | **0.0*** | [0.0 / + 0.15] |
| Platelets | [Gpt/l] | 79 | [44.0 / 115.8] | 66 | [41.3 / 112.3] | **-8*** | [-18.0 / +1.5] | 70 | [41.5 / 122.5] | 73 | [45.5 / 116.5] | **-2^#^** | [-11.5 / +9.0] |
| Fibrinogen | [g/l] | 2.6 | [1.20 / 3.40] | 2.4 | [1.18 / 3.50] | -0.1 | [-0.20 / 0.00) | 2.4 | [1.05 / 3.50] | 2.3 | [1.20 / 3.48] | -0.1 | [-0.10 / +0.10] |
| MARS: Molecular adsorbent recirculating system; SPAD: Single pass albumin dialysis  INR: International normalized ratio  values represent median and interquartile range | | | | | | | | | | | | | |

Table S4: Course and changes of neurological parameters before and after ECAD. Significant changes (p<0.05) within ECAD are marked with *. Significant differences between both devices (p<0.05) were marked with ^#^.

|  |  | MARS | | | | | | SPAD | | | | | |
| --- | --- | --- | --- | --- | --- | --- | --- | --- | --- | --- | --- | --- | --- |
|  |  | pre | | post | | difference | | pre | | post | | difference | |
| HESA |  | 3 | [2.0 / 4.0] | 3 | [2.0 / 4.0] | 0 | [0.0 / 0.0] | 3 | [2.0 / 4.0] | 3 | [2.0 / 4.0] | 0 | [0.0 / 0.0] |
| Glasgow Coma Score | | 14 | [7.0 / 14] | 14 | [6.6 / 14] | 0 | [0.0 / 0.0] | 14 | [4.5 / 15] | 13 | [4.5 / 14] | 0 | [0.0 / 0.0] |
| RAMSAY Score | | 0 | [0.0 / 2.5] | 0 | [0.0 / 3.0] | 0 | [0.0 / 0.0] | 0 | [0.0 / 3.0] | 0 | [0.0 / 3.0] | 0 | [0.0 / 0.0] |
|  | |  |  |  |  |  |  |  |  |  |  |  |  |
|  | |  |  |  |  |  |  |  |  |  |  |  |  |
| **All cross-over cycles** | | without sedative medication (n=49) | | | | | | with sedative medication (n=20] | | | | | |
| HESA | | 2 | [2 / 3] | 2 | [2 / 3] | 0 | [-0.3 / +1.0] | 4 | [4 / 4] | 4 | [4 / 4] | 0 | [0.0 / 0.0] |
| Glasgow Coma Score | | 14 | [14 / 15] | 14 | [12 / 15] | 0 | [-1.0 / 0.0] | 5 | [3 / 6] | 4 | [3 [5.8] | 0 | [-0.8 / 0.0] |
| RAMSAY Score | | 0 | [0 / 0] | 0 | [0 / 0] | 0 | [0.0 / 0.0] | 3 | [3 / 4] | 4 | [4.5] | 0 | [0.0 / 1.0] |
|  | |  |  |  |  |  |  |  |  |  |  |  |  |
|  | |  |  |  |  |  |  |  |  |  |  |  |  |
|  | | | | | | | | | | | | | |
| MARS: Molecular adsorbent recirculating system; SPAD: Single pass albumin dialysis  HESA: Hepatic encephalopathy scoring system  values represent median and interquartile range | | | | | | | | | | | | | |

Table S5: Course and changes of hemodynamic parameters before and after ECAD. Significant changes (p<0.05) within ECAD are marked with *. Significant differences between both devices (p<0.05) were marked with ^#^.

|  |  | MARS | | | | | | SPAD | | | | | |
| --- | --- | --- | --- | --- | --- | --- | --- | --- | --- | --- | --- | --- | --- |
|  |  | pre | | post | | difference | | pre | | post | | difference | |
| sysBP | [mmHg] | 116 | [103.5 / 130.8] | 120 | [110.0 / 133.0] | +4 | [-3.8 / +16.8} | 114 | [103.0 / 127.0] | 118 | [104.0 / 134.0] | +3 | [-11.0 / +19.0] |
| diaBP | [mmHg] | 51 | [48.0 / 60.8] | 56 | [51.0 / 64.0] | +4 | [-3.0 / +10.0] | 50 | [45.0 / 58.0] | 53 | [47.0 / 62.5] | +3 | [-3.5 / +10.5] |
| MAP | [mmHg] | 71 | [65.3 / 82.0] | 75 | [69.0 / 85.0] | +3 | [-3.8 / +10.0] | 71 | [64.0 / 79.0] | 76 | [68.5 / 85.0] | **+4*** | [-3.0 / +11.5} |
| Heart rate | [bpm] | 93 | [85.0 / 107.0] | 95 | [81.5 / 104.8] | 0 | [-8.0 / +9.0] | 96 | [81.0 / 106.5] | 93 | [81.5 / 102.5] | 0 | [-8.0 / +5.5] |
| CVP | [mmHg] | 11 | [8.0 / 13.5] | 10 | [7.0 / 13.0] | 0 | [-4.0 / +0.5] | 10 | [7.0 / 14.0] | 11 | [7.0 / 14.0] | 0 | [-2.0 / +1.5] |
| ScvO2 | [%] | 71.8 | [67.00 / 77.60] | 73.0 | [68.00 / 76.00] | -0.3 | [-4.43 / +5.28] | 74.1 | [67.00 / 77.50] | 71.7 | [65.45 / 78.33] | -1.1 | [-7.00 / +3.20] |
| CI | [l/m2] | 4.5 | [3.95 / 5.44] | 4.4 | [3.74 / 4.98] | +0.1 | [-0.68 / +0.46] | 3.8 | [3.13 / 4.92] | 4.4 | [3.44 / 5.57] | +0.3 | [-0.19 / +1.21] |
| ITBI |  | 1070 | [890.8 / 1374.3] | 1029 | [940.8 / 1208.5] | +6 | [-59.0 / +79.5] | 1133 | [756.0 / 1224.0] | 1095 | [1011.5 / 1270.0] | +95 | [-63-5 / -290.5] |
| ELWI |  | 9 | [6.3 / 12.8] | 8 | [7.0 / 11.0] | +1 | [-1.8 / +1.0] | 9 | [7.0 / 13.0] | 11 | [7.8 / 12.3] | +1 | [-0.5 / +2.3] |
| MARS: Molecular adsorbent recirculating system; SPAD: Single pass albumin dialysis  sysBP: Systolic lood pressure; diaBP: Diastolic blood pressure; MAP: Mean arterial blood pressure; CVP: Central venous pressure; ScvO2 : Central venous saturation of oxygen; CI: Cardiac Index; ITBI – Intrathoracic blood volume index; ELWI: Extravascular lung water index  values represent median and interquartile range | | | | | | | | | | | | | |

Figure S1: Changes in Cytokine levels, separated to the applied ECAD systems. Box plots represent overall values including all 69 performed ECAD cycles, while bounded dots mark changes of each individual ECAD application. (MARS: Molecular adsorbent recirculating system; SPAD: Single pass albumin dialysis; n.s.: non-significant difference)


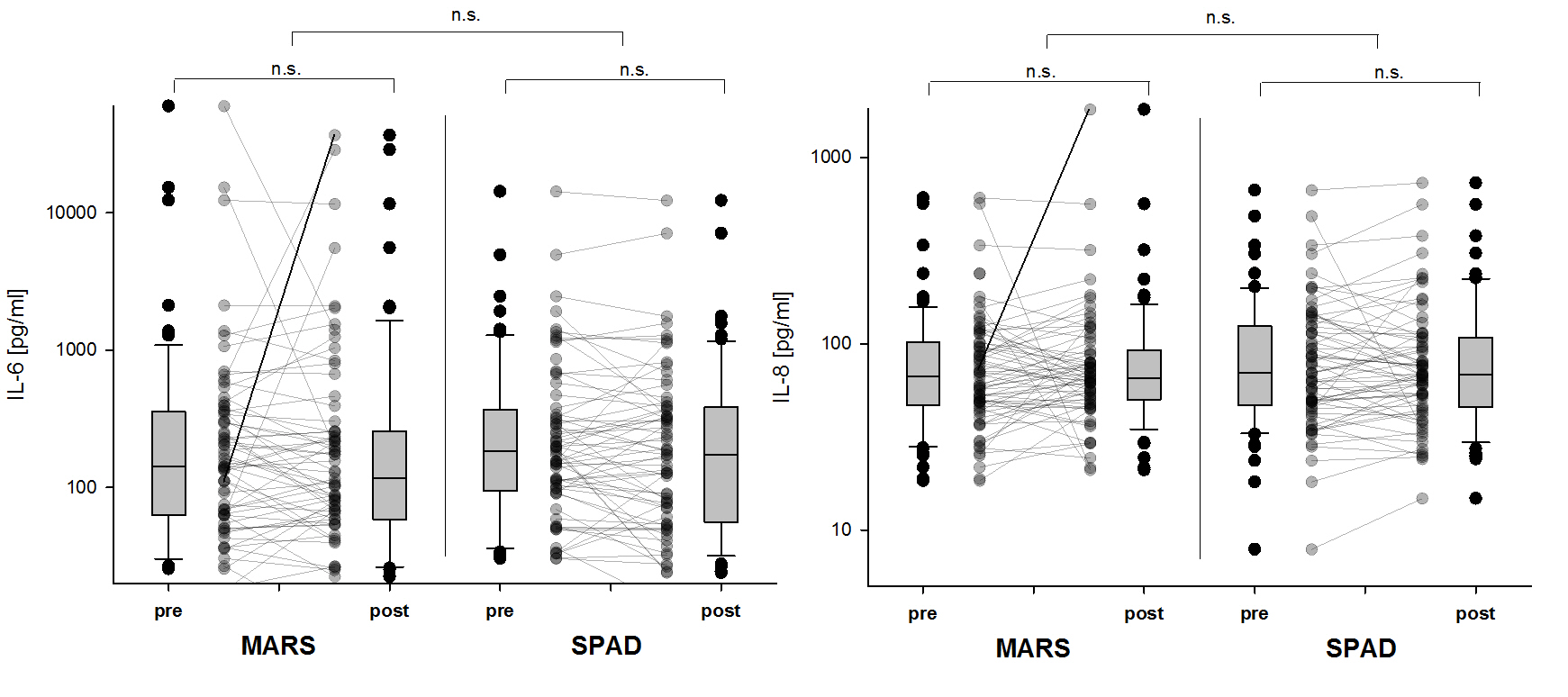

Supplement: Additional file 1: Table S1. — Course and changes of liver enzymes before and after ECAD. Significant changes (p<0.05) within ECAD are marked with *. Significant differences between both devices (p<0.05) were marked with #. Table S2: Course and changes of acid base parameters and electrolytes before and after ECAD. Significant changes (p<0.05) within ECAD are marked with *. Significant differences between both devices (p<0.05) were marked with #. Table S3: Course and changes of blood and coagulation factors before and after ECAD. Significant changes (p<0.05) within ECAD are marked with *. Significant differences between both devices (p<0.05) were marked with #. Table S4: Course and changes of neurological parameters before and after ECAD. Significant changes (p<0.05) within ECAD are marked with *. Significant differences between both devices (p<0.05) were marked with #. Table S5: Course and changes of hemodynamic parameters before and after ECAD. Significant changes (p<0.05) within ECAD are marked with *. Significant differences between both devices (p<0.05) were marked with #. Figure S1: Changes in Cytokine levels, separated to the applied ECAD systems. Box plots represent overall values including all 69 performed ECAD cycles, while bounded dots mark changes of each individual ECAD application. (MARS: Molecular adsorbent recirculating system; SPAD: Single pass albumin dialysis; n.s.: non-significant difference. (DOC 458 kb) [file 13054_2015_1159_MOESM1_ESM.doc]
